# Mutations Affecting Mammalian Aging: GH and GHR vs IGF-1 and Insulin

**DOI:** 10.3389/fgene.2021.667355

**Published:** 2021-11-24

**Authors:** Andrzej Bartke, Holly Brown-Borg

**Affiliations:** ^1^ Department of Internal Medicine, Southern Illinois University School of Medicine, Springfield, IL, United States; ^2^ Department of Biomedical Sciences, University of North Dakota School of Medicine and Health Sciences, Grand Forks, ND, United States

**Keywords:** insulin/insulin-like growth factor signaling, mammalian aging, growth hormone, lifespan, healthspan

**FIGURE 1:**
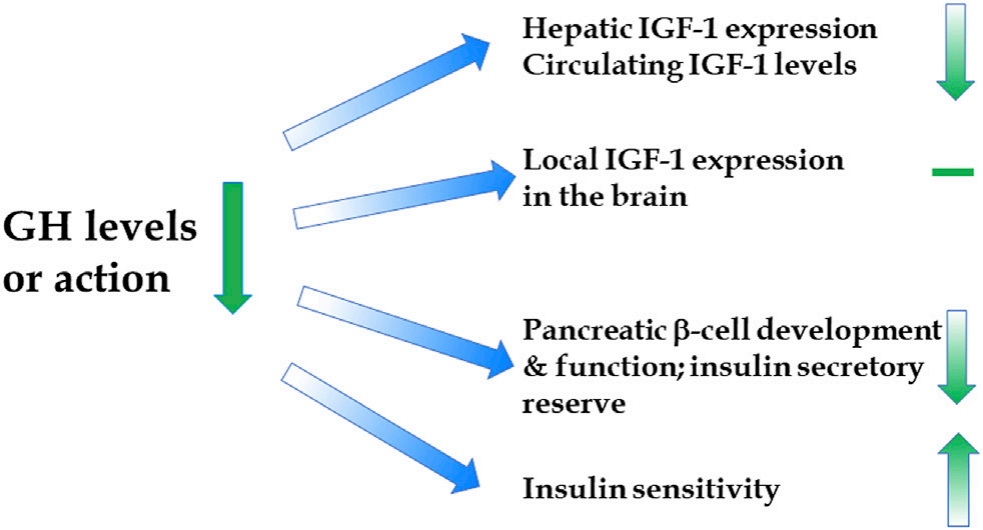
Impact of reduced GH signaling on the levels and actions of IGF-1 and insulin.

**FIGURE 2:**
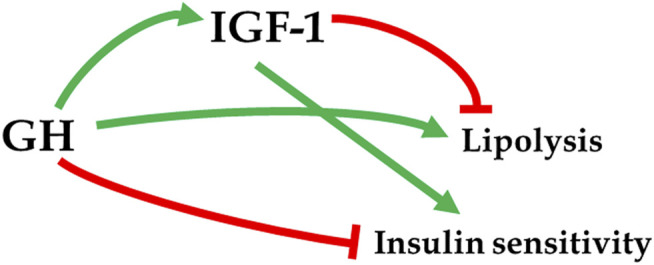
Divergent actions of GH and IGF-1 on metabolic parameters. → stimulation; –| inhibition.

A report of extended longevity in mice homozygous for a mutation producing growth hormone (GH) deficiency (Brown-Borg et al., 1996) was quickly followed by the demonstration of extensive homology between one of the key longevity genes in a worm, Caenorhabditis elegans, and genes coding for insulin and insulin-like growth factor-1 (IGF-1) receptors in mammals (Kimura et al., 1997). Since GH is the key determinant of hepatic IGF-1 expression and circulating IGF-1 levels, and has major impact on insulin signaling (Figure 1), these findings led to an exciting conclusion that the insulin/insulin-like growth factor signaling (IIS) is an evolutionarily conserved mechanism which controls aging in organisms ranging from yeast and worms to insects and mammals. Subsequent work provided much evidence in support of this exciting realization (Tissenbaum and Ruvkun, 1998; Fabrizio et al., 2001; Tatar et al., 2001; Tatar et al., 2003; Piper et al., 2008; Finch and Ruvkun, 2001), and this has led to a focus on IIS, rather than GH signaling, in analyzing genetic control of mammalian aging. *This is an important distinction*. Although biosynthesis and blood plasma levels of GH and IGF-1 are closely linked, the actions of these hormones are not identical and, in some cases, opposite. For example, IGF-1 mimics some of the insulin actions and promotes insulin sensitivity, while GH is anti-insulinemic and promotes insulin resistance; IGF-1 promotes fat deposition, while GH is lipolytic (Figure 2) (Scavo et al., 2004; Veldhuis et al., 2005; Hu et al., 2009). Actions of GH not shared with IGF-1 include other effects relevant to aging such as impact on reactive radicals production and anti-oxidative defenses (Brown-Borg et al., 2002; Bokov et al., 2009), DNA damage and repair (Chesnokova et al., 2019; Chesnokova and Melmed, 2019), macrophage reprogramming (Schneider et al., 2019), ovarian primordial follicle reserve (Saccon et al., 2017), bone resorption and turnover (Thomas and Monson, 2009), kidney dysfunction (Soliman et al., 2019), and cognitive functioning (Nyberg and Hallberg, 2013).

Evidence for the ability of GH to influence healthspan and lifespan of laboratory mice is very strong and includes significant extension of longevity in both sexes of mice with hypopituitarism (combined deficiency of GH, prolactin, and TSH) (Brown-Borg et al., 1996; Flurkey et al., 2001), in mice with isolated GH deficiency due to mutation of Ghrhr gene or deletion of Ghrh (Flurkey et al., 2001; Sun et al., 2013), and in mice with GH resistance due to Ghr gene disruption (Zhou et al., 1997; Coschigano et al., 2003). This evidence for association of genetically reduced GH signaling with extended longevity was obtained in different laboratories and included animals with different genetic background (Bartke and Turyn, 2001; Coschigano et al., 2003; Aguiar-Oliveira and Bartke, 2019). Importantly, extended longevity of hypopituitary Ames dwarf mice can be reduced by GH replacement therapy during the period of rapid peri-pubertal growth (Panici et al., 2010; Sun et al., 2017). This provides evidence that the association of GH deficiency and increased lifespan in Ames dwarf mice is causal (mechanistic).

In contrast to the remarkable extension of longevity in female and male mice lacking GH or GH receptors, the impact of reduced IGF-1 signaling on longevity of IGF1R ± mice and mice treated with an antibody to IGF-1 receptor is modest and seen only in one sex (Holzenberger et al., 2003; Mao et al., 2018; Garratt et al., 2017) (Table 1). This difference between the effects of reduced IGF-1 and GH signaling is likely related to IGF-1 exerting both beneficial and detrimental effects on aging and age-related disease (including opposite effects on the risk of type 2 diabetes vs cardiovascular disease and cognitive decline) and GH having primarily “pro-aging” effects. Both hormones impact growth, but the metabolic effects of GH are significantly greater. Growth hormone has different and more potent effects on glucose regulation when compared to IGF-1. Growth hormone is a regulator of IGF-1 by controlling much of its production and release from the liver and other tissues, and thus regulating plasma concentrations of IGF-1 (Haluzik et al., 2003; Vijayakumar et al., 2011). Liver-derived IGF-1 represents >75% of the circulating hormone (Haluzik et al., 2003; Aguirre et al., 2016). In contrast to the effects on somatic growth, the effects of GH and IGF-1 on glucose homeostasis are markedly different. Growth hormone promotes insulin resistance acting as a counterregulatory mechanism for hypoglycemia (protection during fasting, food deprivation). While GH counteracts insulin action, IGF-1 enhances insulin sensitivity and mimics some of its actions. Both GH and IGF-1 influence insulin production. When GH levels are reduced, insulin levels are also reduced, whereas IGF-1 inhibits insulin secretion (Haluzik et al., 2003). Another complexity is suggested by the evidence that most of IGF-1’s actions on glucose homeostasis and insulin sensitivity are mediated indirectly (through GH suppression), while circulating IGF-1 is bound to high-affinity binding proteins and has low affinity for insulin receptors (Vijayakumar et al., 2011). Direct effects of IGF-1 on glucose management occur mostly in skeletal muscle by increasing glucose uptake (Haluzik et al., 2003; Vijayakumar et al., 2011). Growth hormone influences insulin signaling in liver and adipocytes, whereas no IGF-1 receptors are present in these tissues (Haluzik et al., 2003). Other actions of GH that impact lifespan are also not shared by IGF-1, and thus GH deficiency promotes health and lifespan extension more profoundly than suppression of the levels or action of IGF-1 (Haluzik et al., 2003). Sex-specific responses to suppressing IGF-1 signaling in mice (Ashpole et al., 2017) add to the emerging evidence that, in this species, aging of males is related primarily to the insulin arm of IIS while in females effects of the IGF-1 arm predominate.

**TABLE 1:** Effects of reduced IIS and GH signaling on healthspan and lifespan in different taxonomic groups.

		Yeast	Worms	Insects	Mammals	
					Mice	Humans
IIS	healthspan	?	↑	↑	?	?
	lifespan	↑	↑	↑	↑ (♀ ♀)	—
GH	healthspan	NA	NA	NA	↑	↑
	lifespan	NA	NA	NA	↑	—

In contrast to the findings of extended longevity of IGF-1R heterozygous mice by Holzenberger et al. (Holzenberger et al., 2003), Bokov and his colleagues reported that such animals had very small lifespan extension, no indications of delayed aging, and no changes in end-of-life pathology (Bokov et al., 2011). Discrepancies between the results of a loss of one IGF-1R allele in these two studies were subsequently shown to be related to differences in constitutive IGF-1 signaling and in endocrine responses to reducing the number of IGF-1 receptors in the employed strains of mice (Xu et al., 2014). In further contrast between the effects of suppressing GH and IGF-1 signaling, complete (homologous) disruption of Igf1 or Igf1r genes can have severe detrimental effects on development, postnatal survival and fertility (Liu et al., 1993; Powell-Braxton et al., 1993; Yakar et al., 1999), while GH-deficient and GH-resistant mice are viable and fertile.

Reduced insulin levels and improved insulin sensitivity are associated with extension of longevity in response to calorie restriction or disruption of GH signaling. However, the effects of genetic alterations of insulin levels, global or organ-specific insulin sensitivity, or early steps of intracellular insulin signaling on longevity of laboratory mice are not consistent. Interpretation of the available data is complicated by the negative regulation of expression of the insulin receptors by insulin and by indications that insulin resistance can have both detrimental and protective effects (Barzilai et al., 2012). Templeman and her colleagues reported an 11 percent increase in median longevity of female Ins2^+/−^ Ins1^−/−^ mice in which insulin levels are reduced by approximately 30 percent (Templeman et al., 2017). This association of improved insulin sensitivity and longevity was also seen in other mutants (Masternak et al., 2009; Zhang et al., 2012), but was absent or reversed in others (Shimizu et al., 2011; Nelson et al., 2012; Takeda et al., 2017). Deletion of Insulin receptor substrate 1 (Irs1) extended longevity, but the effects of Irs2 deletion were not consistent in different studies, likely due to difference in the composition of the diet used in the two laboratories (Taguchi et al., 2007; Selman et al., 2008).

Reports of GH signaling and lifespan in rats are very limited. Spontaneous dwarf rats exhibit reduced GH and IIS signaling and longer lifespans compared to controls (Kuramoto et al., 2010; Sasaki et al., 2013). GH-deficient rats generated by antisense GH gene suppression (±) also live 7% longer, but −/− animals do not (Shimokawa et al., 2002). Lewis dwarf rats do not live longer, but are not profoundly GH/IGF-1 deficient (∼55% reduced), exhibit additional endocrine abnormalities (i.e. hyporesponsive HPA axis), and a general tendency towards pro-inflammation resulting in nephropathy and intracerebral hemorrhage, among other issues (Perretti et al., 1993; Oitzl et al., 1995; Sonntag et al., 2005; Ungvari et al., 2010; Groeneweg et al., 2011; Ungvari et al., 2011; Podlutsky et al., 2017).

Collectively, the available evidence suggests that in addition to the evolutionarily conserved role of IIS in the control of aging, GH (which has no known homologs in invertebrates) emerges as a major regulator of aging and longevity in mammals. Alterations in IIS in long-lived GH signaling-related mutants represent some of the multiple mechanisms believed to link GH deficiency or resistance with increases in the healthspan and lifespan (Aguiar-Oliveira and Bartke, 2019).

In humans, the impact of GH and growth/anabolic processes on longevity is more subtle than in laboratory mice, likely reflecting major differences in the pace-of-life including the reproductive strategies (Aguiar-Oliveira and Bartke, 2019; Bartke, 2020). Genetic syndromes of GH deficiency or resistance do not extend human longevity, even though some individuals with these mutations can reach very advanced age (Aguiar-Oliveira and Bartke, 2019). However, pathological excess of GH reduces life expectancy in both humans and mice (Bengtsson et al., 1988; Steger et al., 1993; Wolf et al., 1993) and familial longevity was shown to be associated with reduced GH secretion (van der Spoel et al., 2016). Intriguingly, there is considerable overlap of phenotypic and metabolic consequences of genetic disruption of GH signaling in mice and humans (Aguiar-Oliveira and Bartke, 2019), and humans with these syndromes show a remarkable degree of protection from several age-associated chronic diseases along with indications of extended healthspan, that is “healthy aging” (Guevara-Aguirre et al., 2011; Aguiar-Oliveira and Bartke, 2019).
